# Case mix adjustment of health outcomes, resource use and process indicators in childbirth care: a register-based study

**DOI:** 10.1186/s12884-016-0921-0

**Published:** 2016-05-31

**Authors:** Johan Mesterton, Peter Lindgren, Anna Ekenberg Abreu, Lars Ladfors, Monica Lilja, Sissel Saltvedt, Isis Amer–Wåhlin

**Affiliations:** Medical Management Centre, Karolinska Institutet, Tomtebodavägen 18 A, 171 77 Stockholm, Sweden; Ivbar Institute, Stockholm, Sweden; Departement of Obstetrics and Gynecology, Akademiska Hospital, Uppsala, Sweden; Department of Obstetrics and Gynecology, Institute of Clinical Sciences, Sahlgrenska University Hospital, Gothenburg, Sweden; Department of Obstetrics and Gynecology, Skane University Hospital, Lund, Sweden; Department of Obstetrics and Gynecology, Karolinska University Hospital, Stockholm, Sweden; Department of Women and Child Health, Karolinska Institutet, Stockholm, Sweden; Stockholm County Council, Stockholm, Sweden

**Keywords:** Childbirth, Value-based health care, Case mix adjustment, Outcomes, Caesarean section, Length of stay

## Abstract

**Background:**

Unwarranted variation in care practice and outcomes has gained attention and inter-hospital comparisons are increasingly being used to highlight and understand differences between hospitals. Adjustment for case mix is a prerequisite for meaningful comparisons between hospitals with different patient populations. The objective of this study was to identify and quantify maternal characteristics that impact a set of important indicators of health outcomes, resource use and care process and which could be used for case mix adjustment of comparisons between hospitals.

**Methods:**

In this register-based study, 139 756 deliveries in 2011 and 2012 were identified in regional administrative systems from seven Swedish regions, which together cover 67 % of all deliveries in Sweden. Data were linked to the Medical birth register and Statistics Sweden’s population data. A number of important indicators in childbirth care were studied: Caesarean section (CS), induction of labour, length of stay, perineal tears, haemorrhage > 1000 ml and post-partum infections. Sociodemographic and clinical characteristics deemed relevant for case mix adjustment of outcomes and resource use were identified based on previous literature and based on clinical expertise. Adjustment using logistic and ordinary least squares regression analysis was performed to quantify the impact of these characteristics on the studied indicators.

**Results:**

Almost all case mix factors analysed had an impact on CS rate, induction rate and length of stay and the effect was highly statistically significant for most factors. Maternal age, parity, fetal presentation and multiple birth were strong predictors of all these indicators but a number of additional factors such as born outside the EU, body mass index (BMI) and several complications during pregnancy were also important risk factors. A number of maternal characteristics had a noticeable impact on risk of perineal tears, while the impact of case mix factors was less pronounced for risk of haemorrhage > 1000 ml and post-partum infections.

**Conclusions:**

Maternal characteristics have a large impact on care process, resource use and outcomes in childbirth care. For meaningful comparisons between hospitals and benchmarking, a broad spectrum of sociodemographic and clinical maternal characteristics should be accounted for.

**Electronic supplementary material:**

The online version of this article (doi:10.1186/s12884-016-0921-0) contains supplementary material, which is available to authorized users.

## Background

Most countries have seen rising costs of health care for the past decades and are projected to see continuously increasing health care costs during the coming years, driven by factors such as demographic trends, new technologies and increasing demands [1], but also attributed to substantial unwarranted variations in clinical practice [2]. Considerable attention has been given to how health care must be reformed to meet future demands and budget constraints. One framework for health care management that has gained attention is value-based health care [3]. Within this framework, value is defined as health outcomes achieved in relation to the costs of achieving those outcomes and one proposed mean for achieving higher value in health care is improved transparency [4]. Reporting of health care performance data may affect health care delivery both through patient choice of hospitals who perform well by patients and payers, as well as through changing behaviour among the hospitals [5].

The large volumes in childbirth care and the associated cost, coupled with observed large variations in practices and outcomes, makes childbirth care an extremely relevant area to analyse from a value-based health care perspective. One aspect of childbirth care that has been receiving considerable attention is the use of caesarean section (CS), with increasing rates globally without obvious positive effect on health and with substantial variations between countries and between hospitals [6]. Given that CS are associated with both maternal and neonatal complications and higher resource use [7–10] it is an important indicator to understand value-delivery in childbirth care. However, there are also many other indicators of great relevance for studying care process and value in childbirth care and a number of different indicators have been proposed. For example, induction of labour has been suggested as a quality indicator because of its impact on care process and outcomes and a number of delivery-related complications constitute important aspects of patient-relevant health outcomes [11, 12]. Moreover, length of hospital stay is a fundamental component to understand resource use in relation to childbirth care.

Health care in Sweden is mainly tax-funded with universal coverage. Management of birth in Sweden is relatively standardized, with almost all births taking place in a hospital. All hospitals are staffed by midwives and doctors working together as a team. While women with different risk-profiles are managed at all hospitals, there is a certain degree of specialization with some hospitals managing a higher proportion high-risk births. Because characteristics of patients being treated often differ between hospitals, case mix adjustment for relevant characteristics at baseline is a prerequisite for meaningful comparisons between hospitals. An extensive body of literature is available regarding case mix adjustment of CS rates between hospitals and regions [13–22]. Significantly less has been published regarding risk adjustment of other important indicators related to value in childbirth care. Beyond analyses of CS rates, some studies have performed case mix adjusted comparisons of a set of different indicators [23, 24], while others have focused on single indicators such as perineal tears [25, 26], length of stay [27] and rate of labour induction [28].

The objective of this study was to identify and quantify maternal characteristics that impact a set of important indicators of health outcomes, resource use and care process and which could be used for case mix adjustment of comparisons between hospitals.

## Methods

### Study population and data sources

This register-based study used regional and national databases from 2009 to 2012 to create a unique research database. Women giving birth during 2011 and 2012 were identified in patient administrative systems (PAS) from seven Swedish regions who elected to participate in the Sveus program, which aims at developing systems for value-based monitoring of health care. The participating regions are Jämtland Härjedalen, Östergötland, Dalarna, Uppsala, Skåne, Stockholm and Västra Götaland, which together cover 67 % of all deliveries in Sweden [29]. The PAS are used by the regional health care administrations for analysis, follow-up and reimbursement of care. These databases contain information on diagnoses and procedure codes related to all care consumption in the region. Women giving birth were identified using ICD-10 codes O80-O84 and information related to diagnoses in inpatient care and outpatient specialist care were extracted from PAS from two years prior to admission for childbirth until 12 weeks after admission for delivery. Using record-linkage, data for these women were also extracted from the Medical Birth Register (MBR) [30] and from Statistics Sweden [31]. The MBR, which is largely based on information from the medical charts, was used to capture maternal factors not available in the regional PAS, such as parity, previous CS and body mass index (BMI), while data from Statistics Sweden were used to collect information on country of birth. To allow for complete follow-up, women giving birth in a region different from the one they lived in at the time of delivery, were excluded from analysis. Due to large expected heterogeneity in outcomes and resource use in extremely and very preterm deliveries (deliveries prior to week 32 + 0), these were also excluded from analysis. The regional ethical committee at Karolinska Institutet, Stockholm, Sweden approved the study protocol (Dnr 2013/447-31/5, 2013/1686-32).

### Study variables

The study variables used in the analysis were determined by a cross-professional expert group comprising representatives from professional organizations, regions, and quality registers. Through group discussions during regular meetings, a comprehensive set of variables deemed to be relevant indicators of health outcomes, resource use and care process were identified. In addition, a large number of baseline characteristics with a potential impact on those indicators were identified.

#### Indicators of outcomes, resource use and care process

From the comprehensive list of indicators a limited number of variables of particular relevance for understanding value in childbirth care were selected: The health outcomes for which predictors were assessed were perineal tears of degree 3 and 4 (ICD-10 O70.2-3) in vaginal deliveries and haemorrhage > 1000 ml (ICD-10 O67.8, O72) up to 2 weeks post-partum, as well as post-partum infections up to 12 weeks following admission for delivery, including cystitis (ICD-10 N30, O86.2), endometritis (ICD-10 N71, O85.9) and other delivery-related infections (ICD-10 O86.0,3,4,8, Y95.9). Length of hospital stay was used as an indicator of resource use, and the care process was characterized through the indicators CS (ICD-10 O82, O84.2 or procedure codes MCA00,10,20,30,33,96) and induction of labour (ICD-10 O61 or procedure codes MAC10, DM002, DT027, DT036). Some variables, such as mode of delivery, induction of labour and perineal tears were possible to derive both from PAS and from the MBR and for these variables an analysis was performed to detect possible under-coding in administrative systems.

#### Baseline characteristics

A large number of baseline characteristics deemed relevant for predicting outcomes and resource use were identified based on previous literature and based on clinical expertise. Factors for case mix adjustment differ slightly from risk factors studied extensively in epidemiological literature. The latter may also include factors reflecting the care organization (e.g., type of hospital or delivery ward, hospital size) and interventions during delivery (e.g., mode of delivery, induction of labour, episiotomy, pain relief). While these are extremely important to understand organizational and clinical practice factors that impact outcomes, these should not be controlled for when comparing hospital performance: If these very differences are the underlying reasons for the differences in results, then the inter-hospital comparisons are meant to unveil them and not adjust for them. Hence, only factors that are not a result of the delivery ward’s care process were included, since the objective was to determine the impact of factors which are essentially outside of the delivery ward’s control but which impact care process, resource use and health outcomes:

- Sociodemographic factors (age, born outside the European Union (EU))
- Obstetric characteristics (gestational age, multiple birth, presentation, previous CS, parity and BMI at first prenatal appointment)
- Complications during pregnancy (see full list in Table 1)

**Table 1:** Descriptive statistics of the sample

Sociodemographic factors	Age (mean;sd)	30.7;5.2
	Born outside the EU (%)	21.3
Key clinical factors	BMI (mean;sd)	24.5;4.7
	Nulliparity	44.9 %
	Previous CS	10.0 %
	Non-cephalic presentation	3.5 %
	Multiple birth	1.3 %
	Premature (w32 + 0 - w36 + 6)	4.2 %
Complications during pregnancy	Cervical insufficiency	0.3 %
	Infection of amniotic sac	0.2 %
	Pre-eclampsia	3.4 %
	Post-term pregnancy	5.0 %
	Gestational diabetes	2.0 %
	Polyhydramnios	0.5 %
	Oligohydramnios	1.9 %
	Placenta praevia	0.7 %
	Premature rupture of membranes	1.9 %
	Bleeding during pregnancy	3.0 %
	Herpes	0.6 %
	Intrauterine growth restriction	3.7 %
	Hepatosis	0.8 %
	Placental abruption	0.3 %
Comorbidities	Comorbidity index^a^ (mean;sd)	0.23;0.52
	No comorbidity	81.3 %

^a^The index was calculated as the number of comorbid conditions per patient
- Presence of 14 different groups of comorbidities during 2 years prior to admission for delivery (blood diseases, substance abuse, endocrine and metabolic diseases, gynaecological diseases, heart and vessel diseases, infectious diseases, liver diseases, lung diseases, neurological diseases, renal diseases, mental disorders, musculoskeletal diseases, bowel diseases, tumour diseases).

Detailed definition of all factors are available in Additional file 1: Table S1.

### Statistical analysis

To assess the impact of case mix factors on the indicators of interest, regression analysis was employed using a multivariable regression model with robust standard errors adjusted for clustering of patients within the 21 hospitals in the studied regions. Logistic regression, estimating the odds ratio of each case mix factor, was performed for all dichotomous outcomes. In the case of length of stay in days, ordinary least squares (OLS) regression was used, resulting in a beta coefficient for each case mix factor analysed. The full set of predictors were used in all regression models. To evaluate model fit for the logistic regression models with dichotomous outcomes the c-statistic, calculated as the area under the receiver operating characteristic (ROC) curve, was used. A c-statistic of 0.5 indicates that the model predicts no better than chance alone, whereas a value of 1.0 indicates perfect prediction. R-square was used to determine model fit for the OLS regression model.

## Results

### Study population

After exclusion of extremely and very preterm deliveries (around 1 % of all deliveries) and of women who gave birth in a region different from the one they lived in at the time of delivery (around 3 % of all deliveries) a total of 140 296 deliveries during the study period were identified in administrative systems. For 99.6 % of these a match in the MBR could be identified, resulting in 139 756 deliveries being included in the analysis. Around 5 % of the women did not have a BMI recorded and were consequently excluded from the regression analyses.

### Descriptive statistics

Table 1 presents descriptive statistics on the study population.

The most common comorbidities were mental disorders, neurological diseases and metabolic/endocrine disease, while the frequency of substance abuse, liver diseases and tumour diseases was low. Because of the relatively low frequency of several comorbidities, an index was calculated based on the number of comorbid conditions for each woman to increase robustness of the analysis and to facilitate presentation of the results.

As shown in Table 2, the overall rate of CS and inductions of labour was 17 % and 15 %, respectively. The average length of stay was 2.6 days. Haemorrhage > 1000 ml occurred in 8 % of deliveries. Among post-partum infections, endometritis was most common (2.2 %), followed by other infections and cystitis (1.5 and 0.7 %, respectively). 3.6 % of vaginal deliveries resulted in a degree 3 or 4 perineal tear.

**Table 2:** Care process, resource use and health outcomes

	Care process		Resource use	Health outcomes		
Indicator	CS (%)	Labour induction (%)	Length of stay mean (sd)	Perineal tears^a^(%)	Haemorrhage > 1000 ml^b^(%)	Post-partum infection^c^(%)
Proportion/mean (sd)	16.9	15.0	2.6 (2.0)	3.6	7.8	4.2
n	139 756	139 756	139 756	114 638	137 940	126 387

^a^Perineal tears were studied for women with 84 days follow-up after admission for delivery and who delivered vaginally, ^b^Haemorrhage was studied for women with 14 days follow-up ^c^Infections were studied for women with 84 days follow-up

### Regression analysis

As demonstrated in Table 3, all case mix factors analysed except for premature rupture of membranes were associated with increased risk for CS and most were highly statistically significant. A large number of significant case mix factors were also observed for labour induction, while all case mix factors evaluated were associated with a longer predicted length of stay, all but one statistically significant. The impact of case mix factors on the three adverse health outcomes analysed differed significantly between the different outcomes.

**Table 3:** Impact of case mix factors on the different indicators

	CS	Labour induction	Length of stay (days)	Perineal tears	Haemorrhage > 1000 ml	Post-partum infection
	OR (95 % CI)	OR (95 % CI)	Coefficient (95 % CI)	OR (95 % CI)	OR (95 % CI)	OR (95 % CI)
Age (years)	1.08 (1.07–1.09)	1.03 (1.02–1.03)	0.03 (0.03–0.04)	1.04 (1.03–1.04)	1.03 (1.03–1.04)	1.00 (0.99–1.01)
Born outside the EU	1.15 (1.07–1.24)	1.03 (0.91–1.17)	0.17 (0.09–0.25)	1.15 (1.07–1.24)	1.02 (0.94–1.10)	1.36 (1.28–1.45)
BMI	1.04 (1.04–1.04)	1.04 (1.04–1.05)	0.02 (0.01–0.02)	0.99 (0.98–1.00)	1.01 (1.00–1.01)	1.02 (1.01–1.03)
Nulliparity	4.23 (3.89–4.59)	1.15 (1.03–1.29)	1.35 (1.25–1.46)	6.14 (5.46–6.92)	1.65 (1.55–1.75)	1.84 (1.70–1.98)
Previous CS	20.47 (18.88–22.20)	0.69 (0.63–0.77)	0.73 (0.65–0.80)	5.90 (5.17–6.72)	1.60 (1.46–1.74)	2.40 (2.15–2.67)
Non-cephalic presentation	66.97 (53.39–84.01)	0.25 (0.20–0.32)	0.37 (0.23–0.50)	0.79 (0.33–1.86)	1.02 (0.89–1.17)	1.37 (1.18–1.58)
Multiple birth	3.61 (2.99–4.36)	2.40 (1.81–3.19)	1.45 (1.22–1.69)	0.56 (0.31–1.02)	3.24 (2.79–3.76)	1.76 (1.36–2.27)
Premature (w32 + 0–w36 + 6)	2.02 (1.67–2.45)	0.57 (0.45–0.73)	2.09 (1.81–2.37)	0.22 (0.17–0.30)	0.82 (0.73–0.92)	1.04 (0.90–1.20)
Cervical insufficiency	1.19 (0.85–1.66)	1.39 (0.98–1.99)	0.41 (–0.11–0.94)	0.91 (0.32–2.60)	1.33 (0.96–1.82)	1.62 (1.04–2.52)
Infection of amniotic sac	8.97 (5.19–15.50)	2.58 (1.85–3.61)	1.84 (1.37–2.32)	0.66 (0.34–1.30)	2.11 (1.63–2.73)	3.61 (2.35–5.57)
Pre-eclampsia	2.28 (2.05–2.53)	8.77 (7.30–10.52)	2.19 (1.87–2.52)	1.26 (1.10–1.45)	1.59 (1.38–1.83)	1.29 (1.15–1.46)
Post-term pregnancy	1.80 (1.64–1.98)	17.18 (11.69–25.25)	0.43 (0.36–0.50)	1.52 (1.37–1.68)	1.59 (1.44–1.76)	1.37 (1.25–1.51)
Gestational diabetes	1.69 (1.47–1.95)	2.45 (2.13–2.81)	0.69 (0.48–0.89)	1.27 (1.03–1.56)	1.03 (0.86–1.23)	1.09 (0.92–1.30)
Polyhydramnios	3.37 (2.64–4.30)	3.74 (3.10–4.52)	0.82 (0.56–1.07)	1.62 (0.78–3.35)	1.67 (1.38–2.01)	1.58 (1.25–2.00)
Oligohydramnios	1.68 (1.42–1.99)	19.82 (16.73–23.47)	0.41 (0.33–0.49)	0.85 (0.65–1.11)	1.02 (0.86–1.21)	1.23 (1.05–1.44)
Placenta praevia	9.19 (7.27–11.63)	0.80 (0.60–1.05)	1.42 (0.70–2.14)	1.01 (0.52–1.96)	4.12 (3.38–5.03)	1.76 (1.36–2.29)
Premature rupture of membranes	0.41 (0.33–0.51)	3.26 (2.47–4.29)	0.38 (0.06–0.69)	1.02 (0.73–1.43)	0.85 (0.72–1.00)	1.00 (0.81–1.25)
Bleeding during pregnancy	1.05 (0.95–1.16)	1.40 (1.25–1.57)	0.17 (0.10–0.23)	0.97 (0.81–1.17)	1.14 (1.00–1.31)	1.22 (1.05–1.41)
Herpes	1.12 (0.93–1.36)	1.41 (1.15–1.73)	0.16 (0.05–0.26)	1.01 (0.71–1.43)	0.69 (0.53–0.90)	1.70 (1.31–2.19)
Intrauterine growth restriction	1.33 (1.12–1.58)	2.42 (2.16–2.70)	0.47 (0.26–0.69)	0.66 (0.54–0.81)	0.77 (0.66–0.90)	0.83 (0.67–1.03)
Hepatosis	1.09 (0.91–1.30)	6.68 (5.66–7.88)	0.41 (0.27–0.56)	0.78 (0.51–1.22)	1.31 (1.08–1.60)	0.91 (0.69–1.21)
Placental abruption	46.24 (27.94–76.54)	0.57 (0.46–0.71)	0.43 (0.15–0.71)	2.16 (0.79–5.88)	2.70 (1.86–3.92)	1.76 (1.12–2.78)
Comorbidity index^a^	1.38 (1.34–1.42)	1.35 (1.30–1.41)	0.27 (0.22–0.31)	0.98 (0.93–1.03)	1.12 (1.07–1.18)	1.37 (1.32–1.42)
Constant	0.001	0.014	0.038	0.004	0.017	0.015
n	132 863	132 863	132 863	109 024	131 135	120 072

^a^The index was calculated as the number of comorbid conditions per patient

Higher maternal age increased both length of stay, rates of labour induction, CS, perineal tears and haemorrhage while no effect was observed on infection. Women born outside the EU had higher rates of labour induction and CS, longer length of stay and more complications when controlling for other characteristics. Both nulliparity and previous CS were strongly associated with CS, longer hospital stay and also with higher risk of perineal tears, haemorrhage and post-partum infection. Non-cephalic presentation was a very strong predictor of CS, but had a relatively limited impact on length of stay and risk of complications. Multiple birth had a strong effect on risk of haemorrhage while premature delivery (w32 + 0–w36 + 6) was associated with lower risk of both perineal tears and haemorrhage. Complications during pregnancy were typically associated with higher rates of CS, labour induction and longer length of stay. The complications had a limited impact on the risk of perineal tears while most were associated with increased risk of haemorrhage and post-partum infections.

Maternal comorbidity was also associated with higher intervention rate, longer hospital stay and higher infection and haemorrhage rates but did not have any effect on risk of perineal tears. Analyses were conducted to assess the impact of each single comorbidity on the indicators of interest and with few (mostly non-significant) exceptions the single comorbid conditions were associated with higher rates of CS and labour induction, longer hospital stay and higher risk of haemorrhage and infections. Obviously the different comorbid conditions had slightly different impact on different outcomes. However, forming an index based on the number of comorbidities and using the index instead of the individual comorbidities did not impact the effect of the other variables nor did it have an noteworthy impact on the model fit, a finding in line with previous analyses on comorbidity indices [32].

**Fig. 1:**
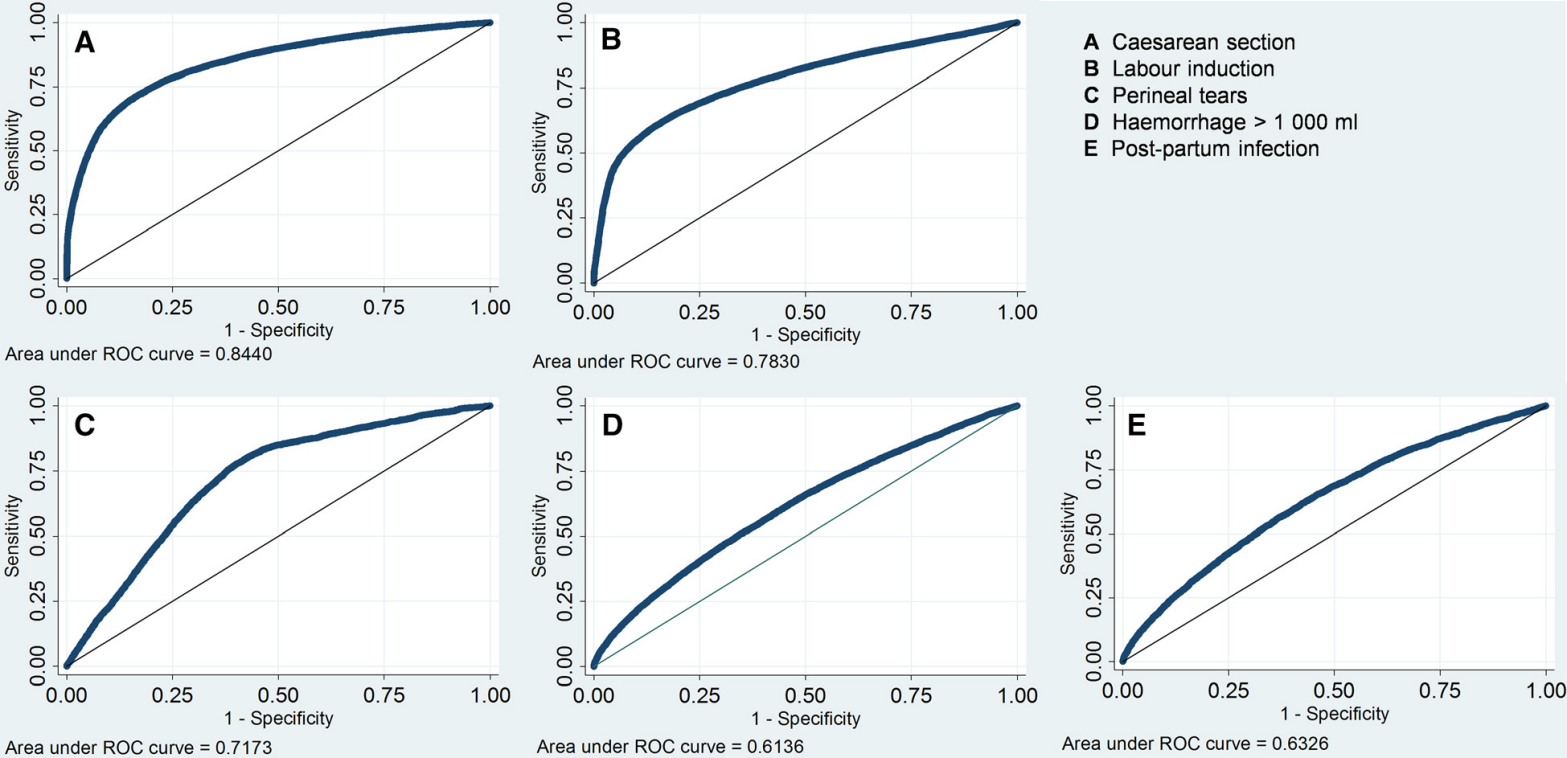
Receiver operating characteristic curves of the five models with dichotomous outcomes

Figure 1 presents ROC curves for the five models with dichotomous outcome variables. The outcome for which the predictive ability was highest was the one for CS (c-statistic 0.84), indicating that the model could reasonably well predict mode of delivery. The predictive ability was also relatively high for labour induction (c-statistic 0.78). Among health outcomes, the predictive ability was reasonable for perineal tears (c-statistic 0.72), where nulliparity and previous CS were two factors contributing to a large portion of the predictive ability. For haemorrhage and post-partum infections (c-statistics of 0.61 and 0.63, respectively) the predictive ability was lower, indicating that the maternal characteristics included had a smaller impact on these two indicators. The regression model of length of stay was able to explain 28 % of the variation between women in length of stay (not presented in figure).

## Discussion

In this study we have performed a comprehensive analysis of case mix factors that are important when analysing hospital performance in childbirth care, one of the most common health care activities. This study assessed the impact of maternal characteristics on a number of different important indicators of care process, resource use and health outcomes in childbirth care. We have demonstrated that a broad spectrum of maternal characteristics should be accounted for when comparing important indicators of value in childbirth care.

One significant strength of this study is that almost 140,000 deliveries were included in the analysis, which allowed for accurate estimation of the impact of maternal characteristics on the outcomes of interest. In addition to the large number of observations, the database used was very comprehensive in terms of follow-up, capturing information from two years before delivery until 12 weeks post-partum. The dataset comprised information not only in relation to the admission for childbirth but all diagnoses in both inpatient and outpatient specialist care. This allowed for a deep understanding of health profile at time of admission and also for estimating rates of complications beyond those identified and diagnosed during the initial hospital admission, which is particularly valuable for studying post-partum infections.

A limitation of this analysis is that many maternal characteristics and outcomes are based on diagnosis codes in PAS and the results are consequently sensitive to appropriate coding of diagnoses and transfer of codes between patient medical record and administrative systems. However, an analysis of possible under-coding of mode of delivery, labour induction and perineal tears showed that the quality of the coding of these diagnosis and procedure codes was generally excellent in the administrative systems compared to the MBR. For CS there was almost a 1:1 match between the MBR and the administrative systems. Nevertheless, the extent to which comorbidities and complications during pregnancy were identified depends on how consistently these are diagnosed and how well these diagnoses are recorded in administrative systems. Some regional differences were observed, indicating possible local variations in definitions of conditions or simply differences in coding practice. One example was diagnosis of gestational diabetes, which varied significantly between regions and hospitals. Differences in screening regimes is likely to explain these large variations [33].

A number of previous studies of case mix adjusted hospital differences of CS rates report the impact of case mix factors. A US study by Glantz et al. [28] estimated similar coefficients to the ones observed in our study for factors such as nonvertex presentation, previous CS, parity, and a number of different complications during pregnancy. Aron et al. [13] included a broad set of clinical predictors and found similar effects on many conditions during pregnancy and obstetric conditions in relation to delivery. In line with our findings, they demonstrated the importance of incorporating pre-existing comorbid conditions in risk adjustment. Bragg et al. [20] studied unadjusted and adjusted differences in CS rates between NHS trusts and found a large number of significant predictors, with breech position, placenta praevia or placental abruption, parity and previous CS being the strongest risk factors. A recent study by Maso et al. [17] also reported strong effects of maternal age, BMI, gestational age and parity on CS rates in an Italian region. Hence, our findings largely corroborate findings from the literature available on risk factors for CS.

In line with our findings, a US study [28] observed preeclampsia, oligohydramnios/polyhydramnios and post-term pregnancy to be strongly associated with labour induction, while previous CS decreased the probability of induction of labour. Because length of stay may be considered an indicator of lower clinical relevance it is perhaps not surprising that its relationship with demographic and clinical risk factors has not received the same attention in the literature. However, length of stay is a critical component from a resource use perspective and in light of scarce resources allocated to health care and the large volumes of childbirth care, benchmarking of length of stay in relation to delivery is highly relevant from a value-based health care perspective. In concordance with a previous study [27] we found that a virtually all obstetric complications and pre-existing medical conditions were associated with longer expected length of stay, indicating that maternal characteristics need to be accounted for in inter-hospital comparisons of this indicator.

A recent US study [24] analysed risk adjustment of very similar obstetric outcomes to the ones studied here. Despite some differences in definitions of these outcomes many findings are in line with those presented here, such as nulliparity and previous CS being strong risk factors of all three adverse outcomes. Their analysis showed that hospital rankings based on the frequency of adverse obstetric outcomes in several cases differed depending on whether unadjusted or case mix adjusted risks were used. In relation to the other outcome indicators, post-partum infection is likely more sensitive to coding differences and differences in care organization, such as to what extent women seek primary care for certain infections. However, it is a very relevant health outcome and an outcome that is not adequately followed up today. By capturing infections both during the childbirth admission and in all inpatient and outpatient specialized care during the 12 following weeks, we believe we have captured infections reasonably well. Nevertheless, further validations of this would be of interest, for example through comparisons to use of antibiotics.

Contrary to most previous studies, we have included age as a linear variable in our analysis. Thanks to the large sample, we could perform analyses of levels of indicators by year of age. For the indicators analysed, a relatively linear impact of age was observed and the predictive ability of the models was lower when exploring modelling the impact of age in categories rather than as a continuous variable. Hence, in order not to lose predictive ability by creating larger age groups, age was included as a continuous variable. Obviously, non-linear effects of age could be included in the predictions. However, analyses showed that the predictive ability of the models was not improved or very marginally improved when including non-linear effect of age. Hence, for the sake of parsimony and for ease of clinical interpretation of results we choose not to incorporate non-linear effects.

The fact that women born outside the EU had higher rates of interventions and complications compared to women born in the EU warrants a comment. Since the effect we found of country of birth was estimated controlling for all other risk factors, the underlying reason for the difference is not obstetric history or other medical factors that were analysed here. Adverse outcomes in women born outside the EU has previously been reported from Sweden and it has been suggested that this may in part be due to miscommunication and language barriers [34]. Born outside the EU is also a proxy for low socioeconomic status which may contribute. Whatever the underlying reason, improved care for this group could have a noticeable impact on overall rates of interventions and complications, given that more than one fifth of the studied women were born outside the EU.

The ability of the model to predict women giving birth with a CS compared to vaginal delivery was high and in line with previously reported figures [17, 20]. The somewhat lower ability of the models to predict which women suffer adverse outcomes has also been described in previous studies [24]. Leung et al. [27] achieved significantly higher predictive ability in their analysis of length of stay compared to our study. However, a major reason for the high predictive ability is likely to be the inclusion of mode of delivery in the prediction model which has a strong effect on length of stay. As argued before, we believe that adjusting for mode of delivery masks important differences between hospitals.

Given the large literature available on the topic of “unwarranted variation” [35, 36], it should not come as a surprise that baseline characteristics do not explain all of the variation observed between patients in our analysis. If the models presented here would have explained all variation between patients this would suggest a complete lack of random variation between patients but more importantly this would also suggest a lack of hospital effect on care process, resource use and outcomes. Rather than being able to predict the exact outcome for every single patient, the objective of these models is to identify factors outside of the hospital’s control which may be accounted for when benchmarking health care hospitals in childbirth care. Given the large number of significant predictors and the ability of the models to predict indicators to a certain extent based on baseline characteristics, case mix adjustment should be considered for a variety of different important indicators of value in childbirth care.

## Conclusion

Our results show that a broad spectrum of maternal characteristics, such as sociodemographic information, obstetric factors and comorbidity, have an impact on important indicators in childbirth care, including care process, resource use and health outcomes. For meaningful comparisons of actual performance between hospitals, and for benchmarking and identification of best practice, a comprehensive set of case mix factors should therefore be accounted for.

## Abbreviations

BMI, body mass index; CS, caesarean section; MBR, medical birth register; OLS, ordinary least squares; PAS, patient administrative systems.

## Additional file

Additional file 1: Table S1. Detailed definitions and data source for all case mix variables included. This table details the exact codes and the data source for the variables used for analysis. (XLSX 10 kb)
